# Irbesartan, an angiotensin II type 1 receptor blocker, inhibits colitis-associated tumourigenesis by blocking the MCP-1/CCR2 pathway

**DOI:** 10.1038/s41598-021-99412-8

**Published:** 2021-10-07

**Authors:** Kensuke Hachiya, Masahiro Masuya, Naoki Kuroda, Misao Yoneda, Junya Tsuboi, Keiki Nagaharu, Komei Nishimura, Takuya Shiotani, Kohshi Ohishi, Isao Tawara, Naoyuki Katayama

**Affiliations:** 1 Department of Haematology and Oncology, Mie University Graduate School of Medicine, Tsu, Mie 514-8507 Japan; 2 Course of Nursing Science, Mie University Graduate School of Medicine, 2-174 Edobashi, Tsu, Mie 514-8507 Japan; 3 Department of Gastroenterology, Saiseikai Matsusaka General Hospital, Matsusaka, Mie 515-8557 Japan; 4 Department of Clinical Nutrition Medical Technology Course, Suzuka University of Medical Science, Suzuka, Mie 510-0293 Japan; 5 Department of Gastroenterology and Hepatology, Mie University Graduate School of Medicine, Tsu, Mie 514-8507 Japan; 6 Department of Transfusion Medicine and Cell Therapy, Mie University Hospital, Tsu, Mie 514-8507 Japan; 7 Faculty of Nursing, Suzuka University of Medical Science, Suzuka, Mie 513-8670 Japan

**Keywords:** Gastroenterology, Gastrointestinal models

## Abstract

The introduction of anti-inflammatory therapies has enabled substantial improvement of disease activity in patients with inflammatory bowel diseases (IBD). However, IBD can lead to serious complications such as intestinal fibrosis and colorectal cancer. Therefore, novel therapies reducing the development of these complications are needed. Angiotensin II (Ang II) promotes tissue inflammation by stimulating the production of monocyte chemoattractant protein-1 (MCP-1) or proinflammatory cytokines. It plays a pivotal role in IBD progression. Although blockade of Ang II has been reported to ameliorate experimental colitis and reduce colorectal cancer risk, the cellular and molecular mechanisms remain poorly understood. Our previous work showed that irbesartan, an Ang II type 1 receptor blocker, reduced the number of C–C chemokine receptor 2-positive (CCR2^+^) monocytic cells in the inflamed pancreas. This study aimed to investigate the possible antifibrotic and antitumour effects of irbesartan using the azoxymethane/dextran sodium sulphate mouse model. Irbesartan suppressed MCP-1 production and the accumulation of Ly6C^+^CCR2^+^ monocytes and fibrocytes in the inflamed colon, downregulated the expression of type 1 collagen and matrix metalloproteinase 9 and inhibited the development of intestinal fibrosis and tumours. Our observations suggest that blocking the MCP-1/CCR2 pathway using irbesartan might be beneficial in preventing colitis-associated colon tumours.

## Introduction

The prevalence of inflammatory bowel diseases (IBD), such as Crohn’s disease and ulcerative colitis, is increasing worldwide. However, the aetiology of IBD remains unclear. IBD can lead to serious complications such as intestinal fibrosis and colorectal cancer (CRC)^1,2^. Colitis-associated CRC is difficult to treat and has a high mortality rate^2,3^. Therefore, it is necessary to investigate the mechanisms of CRC development in the context of chronic inflammation to establish novel therapeutic strategies for IBD patients.

It is well known that chronic continuous inflammation favours the development of fibrosis and tumours. Monocytes and their progenies, which migrate into the inflammatory sites via the monocyte chemoattractant protein-1 (MCP-1)/CC chemokine receptor 2 (CCR2) pathway, play a crucial role in these phenomena^4–9^. Fibrocytes, which differentiate from CCR2^+^ monocytes and express both haematopoietic (CD45) and connective tissue (type I collagen [Col I]) markers, promote tissue fibrosis^10,11^. Tumour-associated macrophages derived from CCR2^+^ monocytes create an immunosuppressive tumour microenvironment to accelerate tumour growth by producing cytokines and chemokines^5^. We previously reported that blocking the MCP-1/CCR2 pathway in the haematopoietic cells ameliorated colitis and prevented intestinal fibrosis during chronic inflammation^12^. The MCP-1/CCR2 pathway has been associated with tumour development and metastasis^13,14^. Although several CCR2 antagonists for cancer therapy are being developed^13–15^, their efficacy and safety remain unclear.

Angiotensin II (Ang II) is the main effector of the renin-angiotensin system (RAS). It regulates blood pressure and salt and fluid balance. Furthermore, Ang II is a proinflammatory hormone that has been involved in many pathological conditions, including IBD^16–20^. Ang II promotes tissue inflammation by stimulating the synthesis and secretion of MCP-1 or proinflammatory cytokines, such as interleukin-6 (IL-6) and tumour necrosis factor-α (TNF-α), in endothelial cells, cancer cells and haematopoietic cells^18,19,21–23^. Ang II type 1 receptor (AT1R) blockers (ARBs), which are extensively used for the treatment of hypertension, are known to downregulate MCP-1 and proinflammatory cytokines and prevent chronic inflammation-associated remodelling in the liver, vessel, kidney and heart^24–27^. In addition, we reported that irbesartan, an ARB, suppressed the in vitro chemotaxis of lymphocyte antigen 6C-positive (Ly6C^+^) monocytes towards MCP-1 and the in vivo migration of adoptive transferred Ly6C^+^ monocytes into the inflamed pancreas^28^. Thus, irbesartan might also have high affinity for CCR2 and might inhibit MCP-1 action beyond the blockade of AT1R. However, it remains uncertain whether ARBs display a therapeutic efficacy in colitis, intestinal fibrosis and colitis-associated CRC.

In this study, we investigated whether irbesartan, which potentially acts as a direct CCR2 antagonist, prevents colitis, intestinal fibrosis and tumourigenesis, in an azoxymethane (AOM) and dextran sodium sulphate (DSS) mouse model of colitis-associated CRC^29^. We found that irbesartan suppressed the production of MCP-1, blocked the recruitment of Ly6C^high^CCR2^+^ inflammatory monocytes to the inflamed colon through the MCP-1/CCR2 pathway and inhibited the development of colitis, fibrosis and tumours*.* Furthermore, irbesartan reversed the tumour progression even after colon tumours were established.

## Results

### Irbesartan ameliorates chemical-induced colitis and tumourigenesis

**Figure 1:**
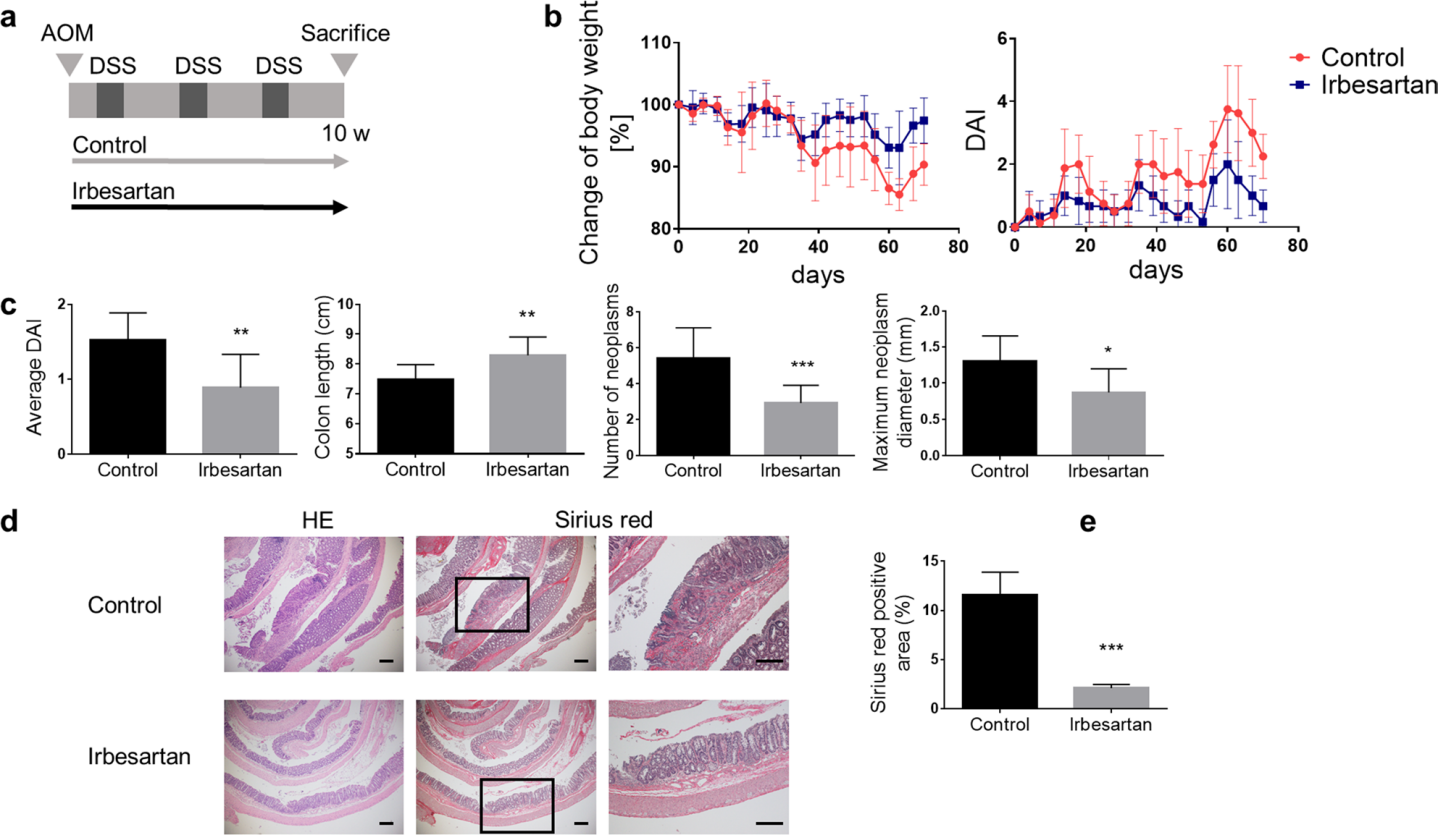
Effect of irbesartan after chronic azoxymethane/dextran sodium sulphate (AOM/DSS) treatment. (**a**) Experimental design. (**b**) The body weight changes and disease activity index (DAI) scores were monitored three times per week during the experimental period in AOM/DSS-treated mice receiving irbesartan (irbesartan-treated mice) or not (control mice). (**c**) The average DAI score obtained during the experimental period and the colon length, number of neoplasms and size of maximum neoplasm, measured 10 weeks after AOM injection, were compared between control and irbesartan-treated mice. The number of mice for each group was 10; pooled data from two experiments. (**d**) Paraffin-embedded colon sections obtained from control (n = 3) and irbesartan-treated mice (n = 3) were stained with haematoxylin and eosin (HE) and Sirius red. The enlarged images of the boxed areas in the centre panels are shown in the right panels. Scale bars, 200 μm. (**e**) Quantification of Sirius red-positive areas. The histograms show the mean percentage of stained area in the total colon area. The experiments were performed at least twice and yielded similar results. Data are expressed as mean ± SD. *P < 0.05; **P < 0.01 and ***P < 0.001 versus control mice.

The efficacy of irbesartan against colitis-associated tumourigenesis was investigated in a well-established model of colitis-associated CRC induced by AOM/DSS treatment in bone marrow (BM) chimeric mice transplanted with BM mononuclear cells from enhanced green fluorescent protein (EGFP)-transgenic mice (EGFP-BM chimeric mice). EGFP-BM chimeric mice receiving AOM/DSS were treated or not with irbesartan (namely, irbesartan-treated mice and control mice, respectively). The protocol is summarised in Fig. 1a. The body weight change after starting the AOM/DSS treatment is presented as the percent change from the baseline value measured at day 0. It was improved in irbesartan-treated mice compared with that of control mice (Fig. 1b). The disease activity index (DAI) score and the colon shortening were significantly lower in irbesartan-treated mice than those in control mice (P < 0.01, Fig. 1c). Furthermore, the neoplasms were significantly fewer and the maximum neoplasm diameter was significantly shorter in irbesartan-treated mice than they were in control mice (P < 0.001 and P < 0.05, Fig. 1c). Histological examination of control mouse colons showed crypt destruction, inflammatory adenomatous formations and fibrosis, which was assessed by Sirius red staining (Fig. 1d). In contrast, in irbesartan-treated mice, the colonic mucosal architecture was preserved, and the fibrosis area was decreased by 81.8% compared with that of control mice (P < 0.001, Fig. 1e). These results suggest that irbesartan improves colitis, fibrosis and colitis-associated tumourigenesis in the AOM/DSS model.

### Irbesartan reduces the accumulation of CCR2^+^ monocytes and fibrocytes in the inflamed colon

**Figure 2:**
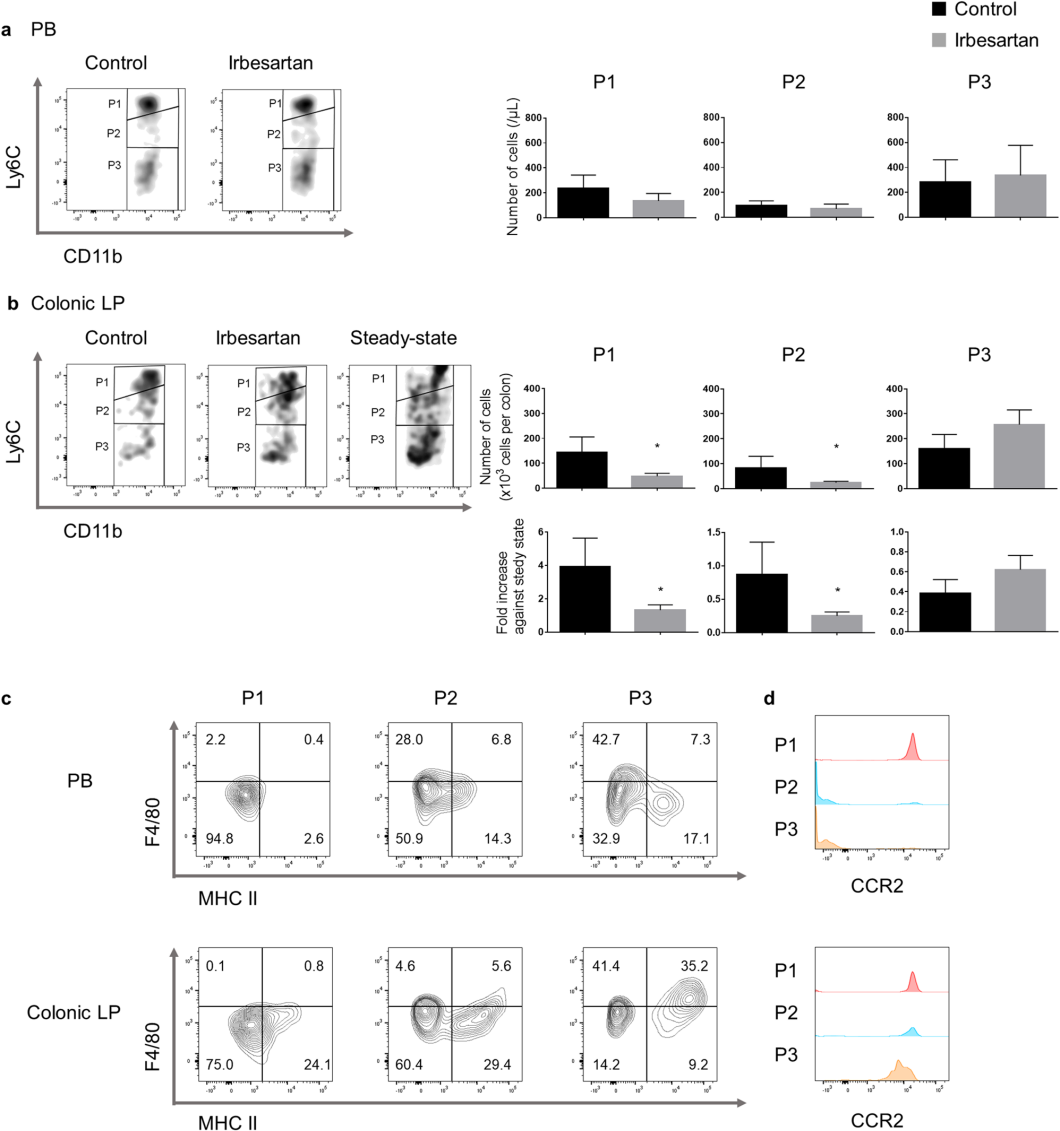
Monocyte-derived cell heterogeneity in the peripheral blood (PB) and colonic lamina propria (LP). (**a**) Flow cytometry representative dot plots of the PB of control and irbesartan-treated mice. Three different monocyte-derived cell fractions (P1, P2 and P3) were defined by the different expression of lymphocyte antigen 6C (Ly6C). The absolute cell numbers for each fraction in the PB of both mice groups are shown. (**b**) Flow cytometry representative dot plots of colonic LP of control, irbesartan-treated and steady-state mice. The absolute cell numbers for each fraction in the colonic LP of control and irbesartan-treated mice are shown in the upper row. The fold increase of each fraction compared to the value at steady state in the colonic LP from control and irbesartan-treated mice appears in the bottom row. Data are presented as mean ± SD (control mice, n = 4; irbesartan-treated mice, n = 4; steady-state mice, n = 4) (**c**) Representative flow cytometry analysis showing the F4/80 and MHC-II-expressing myeloid subpopulations in the three fractions obtained from the PB and colonic LP in control mice. (**d**) Fluorescence-activated cell sorting histograms showing CCR2 expression profiles in the three fractions obtained from the PB and colonic LP in control mice. *P < 0.05 versus control mice.

We quantified haematopoietic cells in the peripheral blood (PB) and colonic lamina propria (LP) using flow cytometry. There were no significant differences in the number of neutrophils, eosinophils, B cells, T cells and NK cells in the PB and colonic LP between control and irbesartan-treated mice (data not shown). As shown in Fig. 2a, b, CD11b^+^ cells, from which Ly6G^+^ neutrophils and sialic acid-binding immunoglobulin-type lectin F-positive (Siglec F^+^) eosinophils were excluded, were subdivided into three distinct monocyte differentiation stages according to Ly6C expression levels (P1, Ly6C^high^; P2, Ly6C^int^; P3, Ly6C^low/neg^). In the colonic LP obtained from control mice, approximately 80% of Ly6C^low/neg^ cells (P3) were positive for F4/80 and 50% of them were positive for the major histocompatibility complex (MHC) II (Fig. 2c), indicating that the P3 fraction corresponded to macrophages. Nearly half of the P3 fraction in the PB from control mice was positive for F4/80 and the P3 fraction was mostly negative for MHC II (Fig. 2c). We compared the presence of these three subpopulations in EGFP-BM chimeric mice not treated with AOM/DSS (steady-state mice), control mice and irbesartan-treated mice. In steady-state mice, the colonic LP contained a large population of macrophages (P3; Fig. 2b). The P1 fraction increased the most (4.0 folds), the P2 fraction was nearly unchanged and the P3 fraction was dramatically decreased by 40% in the colonic LP of control mice after AOM/DSS treatment (bottom row of Fig. 2b). These results were comparable to those previously reported by Soncin et al.^30^. Moreover, we found that the absolute amounts of P1 and P2 fractions were significantly decreased in the colonic LP of irbesartan-treated mice compared with those of control mice, while they were not affected in the PB (Fig. 2a, b). The absolute amounts of the P3 fraction in the PB and colonic LP were not different between control and irbesartan-treated mice (Fig. 2a, b). The amount of P1 fraction in the colonic LP of irbesartan-treated mice was almost the same as that of steady-state mice, while the P2 fraction in the colonic LP was significantly smaller in irbesartan-treated mice than that in steady-state mice (bottom row of Fig. 2b).

**Figure 3:**
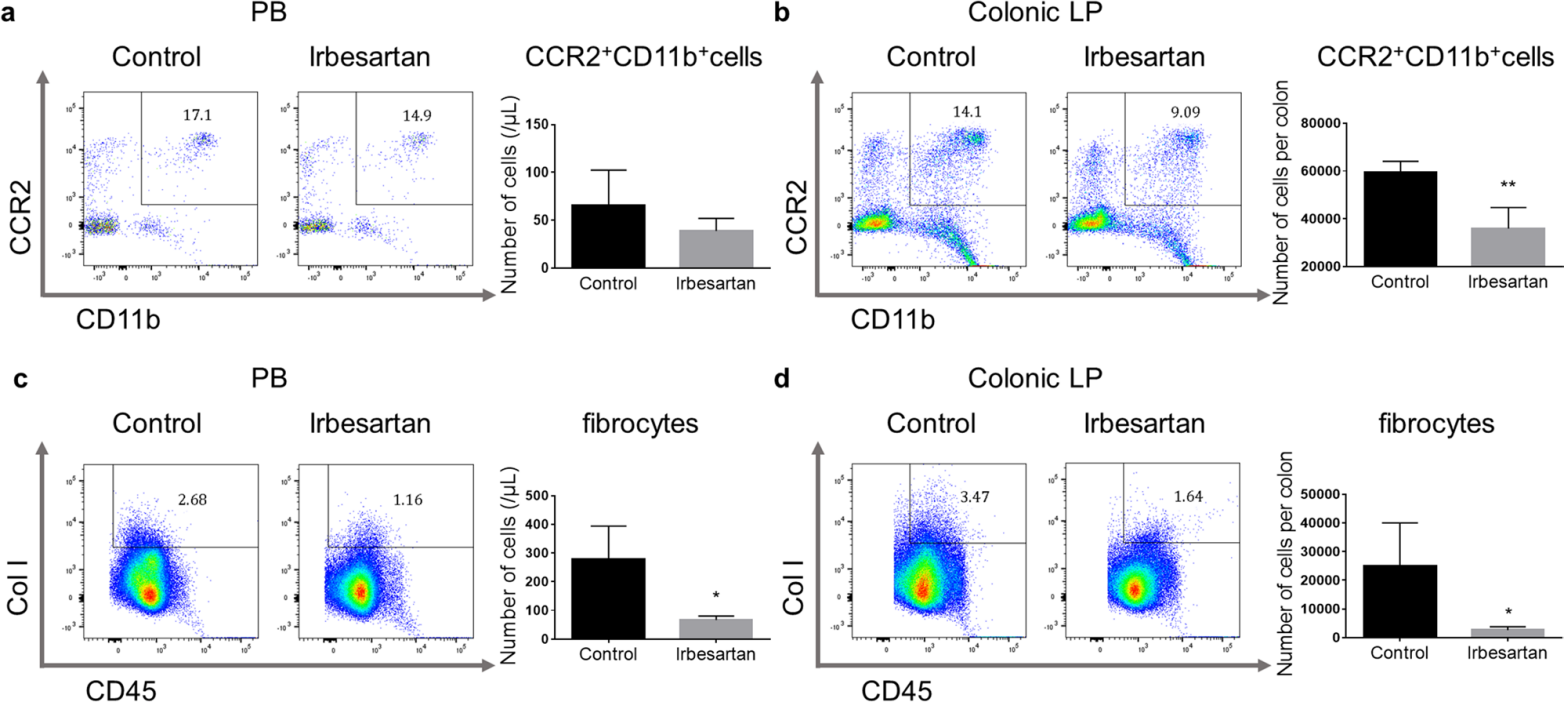
Reduction of CCR2^+^CD11b^+^ cells and CD45^+^Col I^+^ fibrocytes in the peripheral blood (PB) and colonic lamina propria (LP) by irbesartan. (**a**, **b**) Representative dot plots of CCR2^+^CD11b^+^ cells in PB and colonic LP obtained from control and irbesartan-treated mice. Quantification of CCR2^+^CD11b^+^ cells as absolute numbers for each mouse group. (**c**, **d**) Representative dot plots of CD45^+^Col I^+^ fibrocytes in PB and colonic LP obtained from control and irbesartan-treated mice. Quantification of CD45^+^Col I^+^ fibrocytes as absolute numbers for each mouse group. Data are presented as mean ± SD (control mice, n = 4, and irbesartan-treated mice, n = 4). *P < 0.05 and **P < 0.01 versus control mice. CCR2, CC chemokine receptor 2; Col I, type 1 collagen.

In the PB, CCR2 was highly expressed in the P1 fraction, whereas the P2 and P3 fractions were negative for CCR2 (upper row of Fig. 2d). In the colonic LP, CCR2 was expressed in all three fractions, albeit at higher levels in the P1 and P2 fractions (lower row of Fig. 2d). Next, we analysed the effect of irbesartan on the infiltration of CCR2^+^CD11b^+^ cells into the inflamed colon. Compared with control mice, the absolute number of CCR2^+^CD11b^+^ cells was significantly decreased in the colonic LP but not in the PB of irbesartan-treated mice (Fig. 3a, b).

Fibrocytes, which are positive for both CD45 and Col I, are derived from CCR2^+^ monocytes and play a crucial role in inflammation and fibrosis in the heart, lung, liver, kidney and muscle^8,31–34^. We found that irbesartan prevented intestinal fibrosis (Fig. 1e). We previously showed that fibrocytes accumulate in the injured colon of DSS-induced colitis mice through the MCP-1/CCR2 pathway^12^. Therefore, we examined whether irbesartan inhibited the accumulation of fibrocytes in the inflamed colon. The absolute numbers of fibrocytes in the PB and colonic LP were significantly decreased in irbesartan-treated mice compared with those in control mice (P < 0.05, Fig. 3c, d).

These results suggest that irbesartan inhibits the infiltration of CCR2^+^ monocytes from the PB to the injured colon and prevents fibrocyte accumulation in the inflamed colon.

### Irbesartan suppresses the production of MCP-1 in the inflamed colon

**Figure 4:**
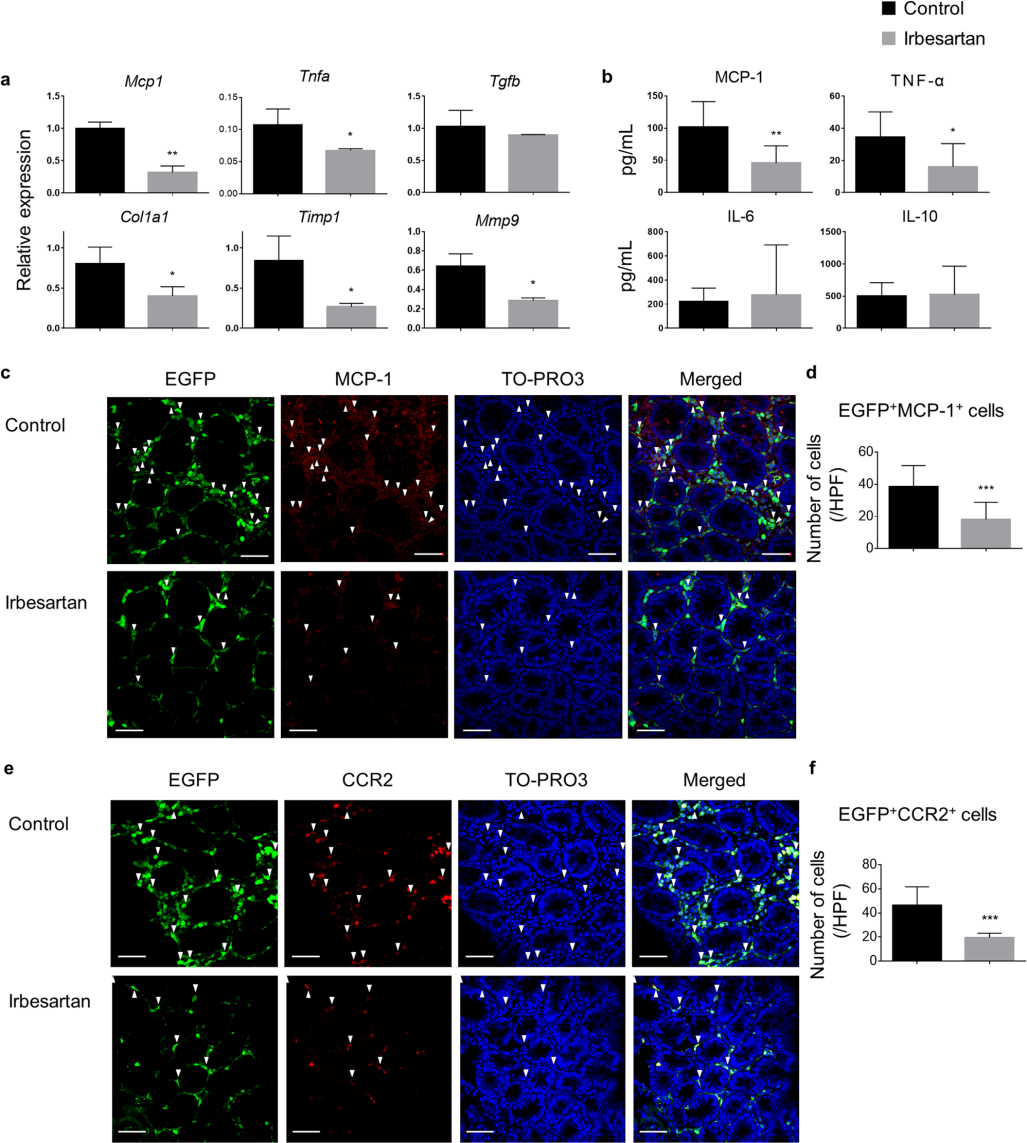
Inhibition of monocyte chemoattractant protein-1 (MCP-1) production in the inflamed colon by irbesartan. (**a**) The histograms show mRNA expression levels relative to that of *Gapdh* of *Mcp1*, *Tnfa*, *Tgfb*, *Col1a1*, *Timp1* and *Mmp9* in colon tissues obtained from control (n = 4) and irbesartan-treated mice (n = 4). The experiments were performed three times and yielded similar results. (**b**) Multiplex assay of chemokine and cytokine plasma concentrations in control and irbesartan-treated mice (n = 8 per group, pooled from two independent experiments). (**c**) Frozen colon sections obtained from control (n = 3) and irbesartan-treated mice (n = 3) 10 weeks after AOM injection. The panels show EGFP in green, MCP-1 in red and TO-PRO3 in blue. White triangles indicate EGFP^+^MCP-1^+^ cells. Scale bars, 50 μm. (**d**) The histograms show the number of EGFP^+^MCP-1^+^ cells in the colon of control and irbesartan-treated mice. (**e**) Frozen colon sections from control and irbesartan-treated mice 10 weeks after AOM injection. The panels show EGFP in green, CCR2 in red and TO-PRO3 in blue. White triangles indicate EGFP^+^CCR2^+^ cells. Scale bars, 50 μm. (**f**) The histograms show the number of EGFP^+^CCR2^+^ cells in the colon of control and irbesartan-treated mice. The experiments were performed twice and yielded similar results. Data are presented as mean ± SD. *P < 0.05; **P < 0.01 and ***P < 0.001 versus control mice.

To investigate the role of irbesartan in mucosal immunity, we evaluated the expression of several genes involved in the inflammatory and fibrotic responses in the rectal tissues and the plasma concentrations of cytokines and chemokines in control and irbesartan-treated mice. The mRNA levels of *Mcp1*, *Tnfa*, *Col1a1*, *Timp1* and *Mmp9* but not *Tgfb* were significantly reduced in rectal tissues from irbesartan-treated mice compared with those of control mice (Fig. 4a). MCP-1 and TNF-α plasma concentrations were significantly lower in irbesartan-treated mice than those in control mice, while there was no difference in the interleukins IL-6 and IL-10 plasma concentrations between the two groups (Fig. 4b). MCP-1 is produced by several cell types, such as macrophages, fibroblasts, endothelial cells, mesangial cells and astrocytes^35–37^. Fluorescence immunohistochemical staining of frozen colon sections from EGFP-BM chimeric mice was performed to investigate the presence of MCP-1-producing EGFP^+^ haematopoietic cells and EGFP^+^CCR2^+^ monocytes/macrophages. Figure 4 c, e show cross sections of colonic crypts. Double immunolabelling revealed that MCP-1 immunoreactivity was mostly detected in EGFP^+^ haematopoietic cells (Fig. 4c). There were many EGFP^+^MCP-1^+^ cells between and around crypts of the colonic LP in control mice. However, in irbesartan-treated mice, the number of EGFP^+^MCP-1^+^ cells was significantly decreased compared with that in control mice (P < 0.001, Fig. 4d). Furthermore, the number of EGFP^+^CCR2^+^ monocytes/macrophages in the colonic LP of irbesartan-treated mice was significantly lower than that of control mice (P < 0.001, Fig. 4e, f). Taken together, these results indicate that some haematopoietic cells, probably macrophages, secrete MCP-1 under inflammatory conditions. Irbesartan attenuates the production of MCP-1 in the colonic LP. This is associated with a reduction of infiltrating CCR2^+^ monocytes/macrophages.

### Irbesartan inhibits MCP-1/CCR2 signalling beyond AT1R blockade

**Figure 5:**
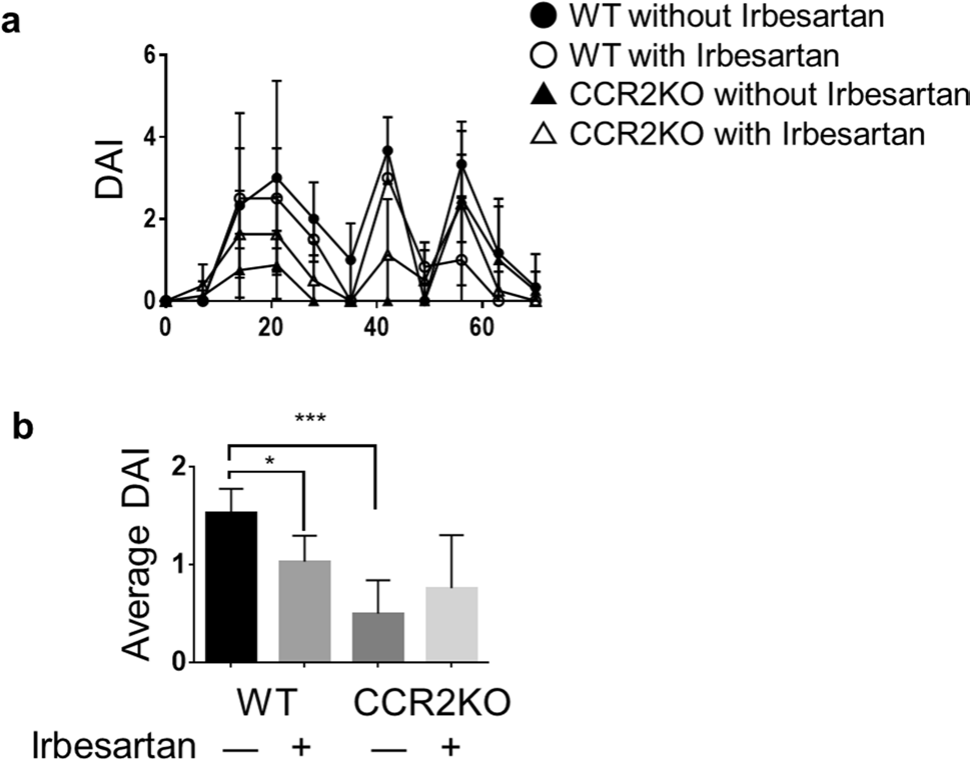
The monocyte chemoattractant protein-1 (MCP-1)/C–C chemokine receptor 2 (CCR2) pathway is the target of irbesartan on colitis-associated colon tumourigenesis. (**a**) The disease activity index (DAI) scores in azoxymethane/dextran sodium sulphate (AOM/DSS)-treated wild-type (WT) and CCR2-deficient mice receiving irbesartan or not were measured three times per week. (**b**) The histograms show average DAI scores obtained during the experimental period. Results were from two independent experiments with eight mice per group (pooled data). The experiments were performed three times and yielded similar results. Data are presented as mean ± SD. *P < 0.05 and ***P < 0.001.

Based on the above observations, we hypothesised that irbesartan ameliorated colitis, fibrosis and colitis-associated tumourigenesis by inhibiting both MCP-1 production and the accumulation of CCR2^+^ monocytes and fibrocytes in the inflamed colon. We previously compared the contribution of CCR2^+^ monocyte-derived cells to chronic DSS-induced intestinal fibrosis by using BM chimeric mice prepared from wild-type (WT) and CCR2-deficient (CCR2^RFP/RFP^) mice. We found that targeted deletion of CCR2 in BM-derived cells attenuated intestinal fibrosis by inhibiting the accumulation of CCR2^+^ monocytes and fibrocytes in the inflamed colon^12^. Ang II-dependent and Ang II-independent anti-inflammatory and antifibrotic effects of ARB have been reported in various organs, such as vessel, kidney, heart and pancreas^14,25–28^. Irbesartan has a higher affinity for CCR2 and inhibits MCP-1 production more strongly than other ARBs^38,39^. Tsukuda et al. demonstrated beneficial effects of irbesartan on ischaemic brain damages beyond AT1R blockade through its inhibitory effects on MCP-1/CCR2 signalling^40^. Therefore, we examined whether irbesartan had a further protective effect on DSS-induced colitis in CCR2^RFP/RFP^-BM chimeric mice. The DAI score was significantly lower in CCR2^RFP/RFP^-BM chimeric mice than that in WT-BM chimeric mice. Irbesartan did not induce a further reduction of the DAI score in CCR2^RFP/RFP^-BM chimeric mice (Fig. 5a, b), indicating that the CCR2-dependent recruitment of inflammatory monocytes is the therapeutic target of irbesartan.

### Irbesartan reduces colitis-associated tumourigenesis during the tumour progression phase after the development of multiple tumours

**Figure 6:**
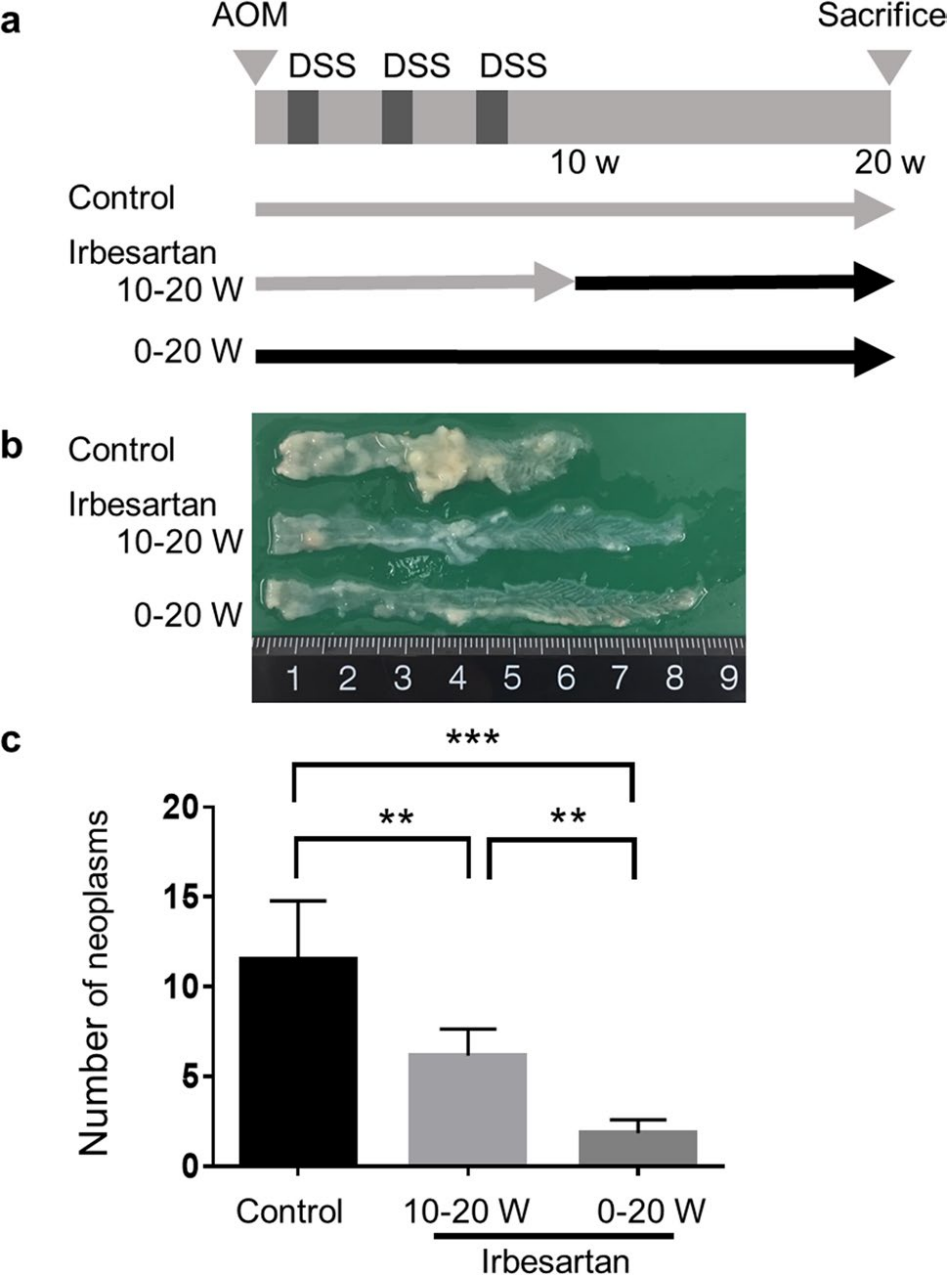
Effect of irbesartan on colitis-associated colon tumour progression. (**a**) Treatment scheme of the azoxymethane/dextran sodium sulphate (AOM/DSS) model. (**b)** Macroscopic view of the colon lumen. Tumours developed in the distal to middle colon of control mice, irbesartan-treated mice for 10–20 weeks and irbesartan-treated mice for 0–20 weeks. Colons were removed 20 weeks after AOM initial administration. (**c**) The histograms show the number of neoplasms. Results are from one experiment with six mice per group. The experiments were performed three times and yielded similar results. Data are presented as mean ± SD. **P < 0.01 and ***P < 0.001.

A growing body of evidence indicates that administrating therapeutic agents before or just after the induction of AOM/DSS treatment minimises inflammation and prevents colitis-associated tumourigenesis^41–44^. However, in clinical trials and practice, the treatment is usually started after the disease has progressed. To investigate the role of irbesartan in the tumour progression phase in our colitis-associated CRC model, we administered irbesartan to WT-BM chimeric mice for 10 weeks after AOM treatment and three cycles of DSS (irbesartan 10–20 W group; Fig. 6a, b). We compared the tumourigenesis in those mice with that in WT-BM chimeric mice no receiving irbesartan (control group) or treated with irbesartan for 20 weeks from the initiation of AOM/DSS treatment (irbesartan 0–20 W group). The number of colon neoplasms in the irbesartan 10–20 W group was highly reduced compared with that in the control group but was significantly greater than that in the irbesartan 0–20 W group (Fig. 6c). Although the number of colon neoplasms in the control group continued to increase after withdrawing DSS, their amount in the irbesartan 10–20 W and irbesartan 0–20 W groups was almost not changed compared with that measured 10 weeks after the start of AOM/DSS treatment (Figs. 1c, 6c). Accordingly, irbesartan inhibited further formation of colon tumours even when it was administered after multiple colon tumours had developed.

## Discussion

The RAS plays an important role in the nervous, cardiovascular and renal systems. Moreover, a local RAS is present in several tissues, such as the pancreas, adipose tissue, gastrointestinal tract, lung and liver^45–49^. The intestinal RAS is thought to be involved in mucosal salt and fluid transfer and in the pathogenesis of colitis. Transgenic mice overproducing active renin are more susceptible to develop experimental colitis^20^. Colonic mucosal levels of renin and Ang II are elevated in IBD patients^16,50^. ARB and angiotensin-converting enzyme inhibitor block the RAS at the receptor or enzymatic levels and have been reported to ameliorate experimental colitis and to reduce CRC incidence, polyp formation and distant metastasis^51–53^. However, their molecular mechanism of action remains unknown.

Tumour-supporting cytokines and chemokines, such as TGF-β, IL-6 and MCP-1, are released from tumoural and stromal cells through AT1R activation by Ang II^23,54^. Several studies have shown that Ang II/AT1R signalling increases the production and release of MCP-1 in vascular smooth muscle cells, monocytes, preadipocytes and osteoblasts^21,55–57^. We recently demonstrated that the mRNA expression of MCP-1 in the colon significantly increases in a time-dependent manner after AOM/DSS treatment. Moreover, blockade of MCP-1/CCR2 pathway in haematopoietic cells by using CCR2^RFP/RFP^-BM chimeric mice ameliorates colitis and prevents the intestinal fibrosis^12^. Irbesartan has a higher affinity for CCR2 and acts as an antagonist to MCP-1^38^. We also previously reported that irbesartan inhibits the in vitro chemotaxis of CCR2^+^Ly6C^+^ monocytes towards MCP-1 and the in vivo migration of adoptively transferred monocytes into carbon tetrachloride-injured pancreas, in which MCP-1 mRNA was highly expressed^28^. Therefore, we examined the therapeutic effects of irbesartan on the development of colitis, intestinal fibrosis and tumourigenesis in the AOM/DSS mouse model of colitis-associated CRC.

Our results demonstrate the following: (1) irbesartan inhibits AOM/DSS-induced colitis, fibrosis and tumourigenesis; (2) this inhibition is associated with a decreased number of CCR2^+^ monocyte-derived cells, including fibrocytes, infiltrated into the inflamed colon and a reduced expression of *Mcp1*, *Tnfa*, *Col1a1*, *Timp1* and *Mmp9* in the colon and (3) irbesartan inhibits the formation of colon tumours even when administered after multiple tumours have developed.

In agreement with our results, Popivanova et al. reported a significantly higher expression of TNF-α and MCP-1 in the inflamed colon of WT mice treated with AOM/DSS. This was associated with a massive intracolonic infiltration of macrophages and the development of multiple colon tumours. The number of tumours was dramatically decreased by the administration of etanercept, a specific TNF-α antagonist, or propagermanium, a CCR2 antagonist, even when given after treatment with AOM and DSS^13,58^. Therefore, the antitumour potential of irbesartan might be associated with the inhibition of TNF-α and MCP-1 signalling.

Fibrocytes are known to play important roles in response to injury or inflammation, tissue remodelling, fibrosis and carcinogenesis^10–12,31–34,59,60^. Fibrocytes secrete paracrine factors, such as platelet-derived growth factor and TGF-β, that activate fibroblasts, enhance the production of extracellular matrix including Col I in digestive cancers and promote cancer proliferation^61^. Matrix metalloproteinases (MMPs) are also produced by fibrocytes^62^. In particular, MMP-9 regulates extracellular matrix remodelling and deposition in the tumour microenvironment and has a crucial role in cancer development and metastasis^63^. Furthermore, fibrocytes in cancer patients are immunosuppressive and may contribute to the immune escape of tumours^64^. We previously reported that targeted deletion of CCR2 in BM-derived cells attenuates colon fibrosis by inhibiting the accumulation of CCR2^+^ monocytes and fibrocytes in the inflamed colon^12^. Herein, irbesartan downregulated the mRNA expression of MMP-9 and Col I, but not of TGF-β, and prevented the tumourigenesis in the colon. Together with our previous data, the present results suggest that the blockade of MCP-1/CCR2 pathway by irbesartan reduces the accumulation of fibrocytes into the inflamed colon and might inhibit tumour progression through the reduction of Col I and MMP-9 production.

There are limitations in the present study. First, we used 30 mg/kg body weight/day of irbesartan and the dose was approximately fivefold higher than the maximum dose for humans. Therefore, further studies are warranted to clarify its effectiveness for murine colitis-related CRC at the standard dose (3 mg/kg body weight) and maximum dose (6 mg/kg body weight) for humans. We did not monitor the changes in blood pressure during the treatment period. In future experiments, we will measure blood pressure in mice to confirm whether the dose of irbesartan is physiologically effective. Second, irbesartan is known to induce greater beneficial effects, which are both dependent on and independent of the AT1R^38–40^. It might bind to CCR2 and block MCP-1 binding. We examined the anti-inflammatory effect of irbesartan on DSS-induced colitis in CCR2^RFP/RFP^-BM chimeric mice. Although the DAI score was decreased in CCR2^RFP/RFP^-BM chimeric mice compared with that in WT-BM chimeric mice, irbesartan treatment did not exert an additive effect in CCR2^RFP/RFP^-BM chimeric mice. This result may indicate the possibility that irbesartan suppressed colonic inflammation through its inhibitory effects on MCP-1/CCR2 signaling. However, we could not clarify whether anti-inflammatory and antitumour effects of irbesartan are mediated by the direct inhibition of Ang II/AT1R signaling. To address this question, we should analyse the role of Ang II/AT1R signaling in AOM/DSS-induced CRC model using Ang II type 1a receptor deficient mice.

In summary, we showed that irbesartan inhibits MCP-1 production and the accumulation of CCR2^+^ inflammatory monocytes and fibrocytes in the inflamed colon and prevents the development of colitis-associated tumours. Therefore, irbesartan might be used as a novel therapeutic strategy for patients with IBD.

## Methods

### Mouse models

The experimental protocol was approved by the Animal Research Committee, Mie University, Japan (approval number: 25-1). All animal experiments were performed in accordance with the institutional guidelines and regulations for animal experiment. The authors complied with the ARRIVE guidelines for all animal experiments. Breeding pairs of C57BL/6J-CD45.1 WT mice and CCR2-deficient mice (CCR2^RFP/RFP^; C57BL/6-Ly5.2 background), in which both CCR2 alleles were replaced by a red fluorescent protein (RFP) sequence, were purchased from Jackson Laboratories (Bar Harbor, ME, USA). Eight-week-old male C57BL/6-Ly5.2 WT mice were purchased from SLC (Shizuoka, Japan). Breeding pairs of EGFP mice (C57BL/6-Ly5.2 background) were kindly provided by Dr. M. Okabe (Osaka University, Japan)^65^. All mice were bred and maintained at the Institute of Laboratory Animals, Mie University, Japan. Ten to 12-week-old male C57BL/6J-Ly5.1 WT mice were irradiated with a single 10-Gy dose of total irradiation using a 4 × 10^6^ V linear accelerator. Then, 5 × 10^6^ BM total nuclear cells obtained from 10-week-old female EGFP, C57BL/6-Ly5.2 WT or CCR2^RFP/RFP^ mice were injected into irradiated male C57BL/6J-CD45.1 WT mice. AOM/DSS treatment started 8 weeks after BM transplantation. At the start of the injury, the mean body weight of BM chimeric mice in both control and irbesartan-treated group was approximately 22 g. Mice were intraperitoneally injected with 10 mg/kg AOM (Wako Pure Chemical Industries, Osaka, Japan) and fed with chow containing irbesartan (30 mg/kg/day, Tokyo Chemical Industry, Tokyo, Japan) or normal chow (control). One week after AOM treatment, mice were given 1% DSS (MW 36–50 kDa, MP Biochemicals, Santa Ana, CA, USA) dissolved in the drinking water for 7 days, followed by water alone for 2 weeks. The DSS treatment was repeated for three cycles. AOM/DSS-induced colitis was scored by DAI, which assesses the weight loss, stool consistency and bleeding^66^. The DAI score was measured three times per week. Mice were euthanised by cervical dislocation after anaesthesia with isoflurane 10 or 20 weeks after AOM injection.

### Tissue preparation

Mice colons were harvested and fixed with 4% phosphate-buffered paraformaldehyde for 1 h at room temperature as previously reported^12^. Some tissue blocks were embedded in paraffin after dehydration in a graded alcohol series. Other tissue blocks were fixed in 4% phosphate-buffered paraformaldehyde, embedded in Tissue-Tek OCT medium (Sakura Finetek USA, Torrance, CA, USA), rapidly frozen by plunging into liquid nitrogen and stored at − 80 °C. Tissue blocks were cut into 5-μm-thick sections using a microtome or a cryostat.

### Histological analysis

Serial sections were stained with haematoxylin and eosin (HE) and Sirius red to evaluate the presence of inflammation, fibrosis and tumours^12^. Three images per section were captured at 100 × magnification using an Olympus BX41 microscope (Olympus, Tokyo, Japan) equipped with a 10 × /0.40 numerical aperture objective lens and an Olympus Camedia C-5060 camera. The percentage of Sirius red staining, which indicated the presence of collagen fibres, was evaluated for the whole area of each image using the ImageJ software (NIH, Bethesda, MD, USA).

### Immunohistochemical analysis

Frozen colon sections were treated with 0.5% Triton X-100 in Ca^2+^- and Mg^2+^-free phosphate-buffered saline (PBS^−^) for 1 h and with blocking reagents containing 3% bovine serum albumin (BSA) for 1 h. Afterwards, they were incubated with goat polyclonal anti-MCP-1 antibody (Santa Cruz Biotechnology, Dallas, TX, USA), followed by incubation with Alexa Fluor 568-conjugated donkey anti-goat IgG (Molecular Probes Invitrogen, Carlsbad, CA, USA) or phycoerythrin-conjugated monoclonal anti-CCR2 antibody (R&D Systems, Minneapolis, MN, USA). Nuclei were stained with TO-PRO3 iodide (Life Technologies, Eugene, OR, USA). Staining without anti-MCP-1 antibody, followed by incubation with Alexa Fluor 568-conjugated donkey anti-goat IgG or with phycoerythrin-conjugated rat IgG2b (BioLegend, San Diego, CA, USA), was performed as a negative control. The sections were examined using an Olympus IX81 FV1000 laser scanning confocal microscope.

### Isolation of PB cells and colonic LP cells

PB cells and colonic LP cells were isolated as previously described^12^. Blood was collected from anaesthetised mice via cardiac puncture using a heparinised syringe. After red blood cell removal using ammonium-chloride-potassium lysis buffer, white blood cells were pelleted by centrifugation at 500×*g* for 10 min at room temperature and resuspended in 400–600 μL of PBS^−^ containing 0.1% BSA. The colons were resected, opened longitudinally and washed with saline to remove intestinal contents. Next, they were cut into 1.0-cm pieces, which were incubated with Hanks’ balanced salt solution (HBSS) [lacking Ca^2+^ and Mg^2+^ and containing 2.5% foetal calf serum (FCS), 1 mM dithiothreitol and 1% penicillin/streptomycin/glutamine] in shaking conditions (200 rpm) at 37 °C for 20 min to remove the mucus. Subsequently, epithelial cells were removed through incubation with HBSS containing 2.5% FCS, 1 mM ethylenediaminetetraacetic acid (Invitrogen, Carlsbad, CA, USA) and 1% penicillin/streptomycin/glutamine, shaking (200 rpm) at 37 °C for 30 min. The latter procedure was performed twice. The colonic pieces were then digested in HBSS containing 2.5% FCS, 1.5 mg/mL collagenase VIII (Sigma-Aldrich, St. Louis, MO, USA) and 0.1 mg/mL DNase I (Worthington Biochemical, Lakewood, NJ, USA) by shaking (200 rpm) at 37 °C for 30 min. The resultant cell suspensions were sequentially passed through cell strainers (70 μm), resuspended in 40% Percoll (GE Healthcare UK, Little Chalfont, UK) and layered on top of 75% Percoll following centrifugation at 2500 rpm for 20 min at room temperature. Cells residing at the interface between two Percoll layers were collected, washed twice with PBS^−^ and resuspended in 0.1% BSA PBS^−^ for use in further experiments.

### Flow cytometry analysis

Isolated cells were incubated with anti-mouse CD16/CD32 (BioLegend) to block non-specific Fc receptors. Then, the cell surface was stained with the corresponding mixture of fluorescently labelled monoclonal antibodies against B220, CD3, CD11b, CD45, F4/80, MHC II (IA/IE), Ly6C, Ly6G, Siglec F (BioLegend), NK-1.1 (Miltenyi Biotec, Auburn, CA, USA) and CCR2. The lineage cocktail consisted of antibodies targeting B220, CD3, Ly6G, NK1.1 and Siglec F. Seven-amino actinomycin D (BioLegend) was used to discriminate live and dead cells. Isolated cells were first gated on size, singularity and positive expression of EGFP and CD45. Next, lineage- and seven-amino actinomycin D-positive cells were eliminated. To stain the intracellular antigens after surface labelling, cells were fixed and permeabilised with Cytofix/Cytoperm kit (BD Biosciences, San Diego, CA, USA) and sequentially incubated with rabbit anti-collagen type I (Rockland, Limerick, PA, USA) and Alexa Fluor 647-conjugated goat anti-rabbit IgG (Invitrogen). Data were acquired on LSRFortessa (BD Biosciences) and processed using FlowJo software (Tree Star, Ashland, OR, USA) with the appropriate isotype controls to determine the gating.

### Analysis of gene expression

Total RNA was extracted from rectum samples using RNeasy Mini Kit (Qiagen, Hilden, Germany) and converted to complementary DNA using the SuperScript III First-Strand Synthesis System for RT-PCR (Invitrogen) according to the manufacturer’s instruction. Quantitative real-time PCR analysis of the complementary DNA was performed with a StepOnePlus Real-Time PCR System Upgrade (Applied Biosystems, Carlsbad, CA, USA) using the default settings. The following primers were used: *Mcp1* (Mm00441242_m1), *Tnfa* (Mm00443259_g1), *Tgfb1* (Mm01178820_m1), *Col1a1* (Mm00801666_g1), *Timp1* (Mm013-41361_m1), *Mmp9* (Mm00442991_m1) and *Gapdh* (Mm99999915_g1). GAPDH gene was amplified as internal control.

### Cytokine quantification

Cytokine levels in mouse plasma samples were determined using flow cytometric bead-based multiplex assays, LEGENDplex Mouse Inflammation Panel (BioLegend), following the manufacturer’s protocol. Briefly, the plasma samples were diluted twice with assay buffer and incubated with the mixed beads for 2 h at room temperature, shaking. Next, they were incubated with detection antibodies for 1 h. Without washing, they were then incubated with streptavidin–phycoerythrin conjugate for 30 min. Finally, the samples were washed and suspended in 200 μL of wash buffer. Data were acquired on BD LSRFortessa and analysed using the BioLegend’s LEGENDplex Data Analysis Software (BioLegend).

### Statistics

Data are expressed as means and standard deviations. Two experimental groups were compared using unpaired two-tailed Student’s *t*-test for two group comparisons. One-way or two-way analysis of variance with Tukey’s multiple comparison test was used for comparisons among three or more groups. Analyses were performed using the Prism software (GraphPad Software, La Jolla, CA, USA). A P value < 0.05 was considered statistically significant.

## Data Availability

The data sets generated during and/or analysed during the current study are available from the corresponding author on reasonable request.
